# 

**Published:** 1877-04

**Authors:** 


### CONTENTS:

ARTICLE I.—Address delivered before the Society of Alumni of the Baltimore College of Dental Surgery. By W. W. H. Thackston, M. D., D. D. S. 529

ARTICLE II.—The Annual Meeting of the Alumni Association of the Baltimore College of Dental Surgery ..... 541

ARTICLE III—Celluloid ..... 545

ARTICLE IV.—The Thirty-Seventh Annual Commencement of the Baltimore College of Dental Surgery ..... 548

ARTICLE V.—The Fourth Annual Commencement of the Maryland Dental College 551

ARTICLE VI.—Pulp Capping. By W. C. Barrett, D. D. S. ..... 552

ARTICLE VII.—Bacteria and Fermentation ..... 555

ARTICLE VIII.—On Dentine. By Chas. Sibler, M. D ..... 558

ARTICLE IX—A Strange Case. By J. Clark Scott, D. D. S ..... 562

ARTICLE X.—Lupus of the Mouth and Pharynx. By G. Homolle ..... 564

ARTICLE XI.—The Results of Nerve Injury ..... 566

ARTICLE XII.—Phosphate of Lime as a Therapeutic Agent ..... 567

ARTICLE XIII.—Psoriasis of the Tongue. By Dr. Nedopil ..... 569

### EDITORIAL, ETC.

Southern Dental Association ..... 570

Professional Inventions ..... 571

A New Medical Journal ..... 572

### MONTHLY SUMMARY.

Partial Exsection of both Upper Jaws for Enchondroma ..... 572

Ununited Fracture of the Forearm, and Treatment by Excision and the Wire Suture ..... 574

Venous Pulse an Habitual Symptom of the Physiological Action of Chloroform .. 576

